# Anabolic Properties of Mixed Wheat-Legume Pasta Products in Old Rats: Impact on Whole-Body Protein Retention and Skeletal Muscle Protein Synthesis

**DOI:** 10.3390/nu12061596

**Published:** 2020-05-29

**Authors:** Insaf Berrazaga, Jérôme Salles, Karima Laleg, Christelle Guillet, Véronique Patrac, Christophe Giraudet, Olivier Le Bacquer, Marine Gueugneau, Philippe Denis, Corinne Pouyet, Angelique Pion, Phelipe Sanchez, Yves Boirie, Valérie Micard, Stéphane Walrand

**Affiliations:** 1 UNH, Unité de Nutrition Humaine, CRNH, Université Clermont Auvergne, INRA, Auvergne, 63000 Clermont-Ferrand, France; berrazaga.insaf@gmail.com (I.B.); jerome.salles@inrae.fr (J.S.); karimalaleg@gmail.com (K.L.); christelle.guillet@uca.fr (C.G.); veronique.patrac@inrae.fr (V.P.); christophe.giraudet@inrae.fr (C.G.); olivier.le-bacquer@inrae.fr (O.L.B.); marine.gueugneau@inrae.fr (M.G.); philippe.denis@inrae.fr (P.D.); corinne.pouyet@inrae.fr (C.P.); angelique.pion@inrae.fr (A.P.); phelipe.sanchez@inrae.fr (P.S.); yves.boirie@inrae.fr (Y.B.); 2 IATE Agropolymer Engineering and Emerging Technologies, University Montpellier, INRA, CIRAD, Montpellier SupAgro, 34060 Montpellier, France; 3 Service de Nutrition Clinique, Centre Hospitalier Universitaire (CHU) Gabriel Montpied, 63000 Clermont-Ferrand, France

**Keywords:** sarcopenia, skeletal muscle, legume-enriched pasta, faba bean, lentil, split pea, protein quality, muscle protein synthesis rate

## Abstract

The mechanisms that are responsible for sarcopenia are numerous, but the altered muscle protein anabolic response to food intake that appears with advancing age plays an important role. Dietary protein quality needs to be optimized to counter this phenomenon. Blending different plant proteins is expected to compensate for the lower anabolic capacity of plant-based when compared to animal-based protein sources. The objective of this work was to evaluate the nutritional value of pasta products that were made from a mix of wheat semolina and faba bean, lentil, or split pea flour, and to assess their effect on protein metabolism as compared to dietary milk proteins in old rats. Forty-three old rats have consumed for six weeks isoproteic and isocaloric diets containing wheat pasta enriched with 62% to 79% legume protein (depending on the type) or milk proteins, i.e., casein or soluble milk proteins (SMP). The protein digestibility of casein and SMP was 5% to 14% higher than legume-enriched pasta. The net protein utilization and skeletal muscle protein synthesis rate were equivalent either in rats fed legume-enriched pasta diets or those fed casein diet, but lower than in rats fed SMP diet. After legume-enriched pasta intake, muscle mass, and protein accretion were in the same range as in the casein and SMP groups. Mixed wheat-legume pasta could be a nutritional strategy for enhancing the protein content and improving the protein quality, i.e., amino acid profile, of this staple food that is more adequate for maintaining muscle mass, especially for older individuals.

## 1. Introduction

Sarcopenia is a syndrome that is characterized by a progressive and generalized loss of skeletal muscle mass and function with a risk of adverse effects, such as physical disability, metabolic abnormalities, poor quality of life, and increased risk of death [1]. The mechanisms that are responsible for sarcopenia are numerous and still only partly understood, but the decreased muscle anabolic response to meal-associated amino acids and insulin that appears during aging plays a key role [2,3,4,5]. In order to overcome this ‘anabolic resistance’ phenomenon [6] and mitigate age-related muscle loss, it is recommended to increase protein intake for older people to 1–1.2 g/kg bodyweight/d [7,8] and optimize the quality of dietary protein intake to meet the needs of older people [9].

There is mounting evidence that proteins that are derived from animal sources, i.e., meat, egg, milk and its constitutive proteins (casein and whey proteins), are strong enhancers of skeletal muscle protein synthesis rate (for reviews, see [10,11,12]). Nevertheless, older people generally intake less animal products, due to reduced chewing efficiency, cognitive dysfunction, blunted appetite for rich protein foods, and/or socio-economic factors. Moreover, excessive consumption of animal-based foods might increase the risk for cardiovascular diseases, chiefly due to high intake of saturated fat [13,14,15]. Therefore, it is necessary to find new protein sources that are adapted for older people. Plant-source proteins could be a valuable strategy for older people to prevent the health risks that are associated with consuming animal products and promote better protein consumption, as plant-based protein sources are rich in fiber and micronutrients and have antioxidant properties [16,17].

Several studies have evaluated the effect of plant-based protein sources, i.e., raw faba beans [18,19,20,21], raw peas, cooked lentils or cooked beans [22,23,24,25,26], soy proteins [27,28,29,30,31,32,33,34,35], faba bean proteins [36], and wheat proteins [33] when compared with animal-based protein sources on body protein retention and metabolism in young or old rats, pigs, or humans. All have reported a positive effect of animal proteins on protein metabolism in young animals or humans as compared to plant-based protein sources, regardless of their form, e.g., raw or cooked, protein isolate, or protein hydrolysate. Yang et al. [37] showed that muscle protein synthesis rate was 40% lower in elderly subjects after soy protein intake than after ingesting the same amount of whey protein. Gorissen et al. [38] also showed that the muscle protein synthesis rate was 30% lower in older men after ingesting 35 g of wheat protein than after ingesting an equal amount of casein. Overall, regardless of age, most of these studies found that plant-based protein sources have less potential to enhance protein metabolism and retention rate at the whole-body or muscle level in animals and humans when compared to animal-based proteins. This difference could be explained by the fact that plant proteins are less digestible [39] and relatively less rich in essential and biologically-active amino acids, like leucine than animal proteins [11]. Plant-based proteins are also known to be deficient in certain essential amino acids (EAA), like lysine, methionine, threonine, and/or tryptophan [40], which could limit in vivo protein synthesis.

Blending different plant-based proteins could theoretically compensate for the lower anabolic potential of these single protein sources [11]. Combining various plant-based protein sources, such as cereals (deficient in lysine and threonine) and legumes (deficient in sulfur amino acids), could provide a more balanced amino acid profile to meet body needs, especially in EAA [41]. In a study on young rats, Márquez-Mota et al. [42] evaluated the effect of cereal and legume protein blends, i.e., corn protein isolate–soy protein isolate and corn protein isolate–black bean protein concentrate, on the anabolic signaling pathway that is involved in protein synthesis. Overall, they demonstrated that plant protein blends had greater effect than a single plant protein source. Torres et al. [43,44,45], Laleg et al. [46], and Giménez et al. [47] also demonstrated the beneficial effects of incorporating 10%–35% legume flour in wheat pasta and 30% legume flour in corn pasta on protein digestibility and net protein utilization (NPU) in young rats. However, to our knowledge, the nutritional benefits of such combinations in older individuals has never yet been studied. 

The aim of this study was to evaluate the efficiency of plant-based protein sources, i.e., wheat pasta enriched with different legume flours (faba bean, lentil, or split pea), on protein digestibility and metabolism, i.e., protein retention, muscle protein synthesis rate, and muscle protein accretion as compared to animal-based dietary proteins, in old rats. For this purpose, we prepared wheat pasta enriched with faba bean, lentil, or split pea flour, as per the classical steps of industrial pasta production (hydration, mixing, extrusion, drying) [48], and then cooked and dried the pasta at low temperature to produce a material that was fed to the rats. We evaluated the protein quality indexes of each legume-enriched pasta, and compared body composition, liver, and skeletal muscle protein contents and skeletal muscle protein synthesis rate in old rats fed legume-enriched pasta or high-nutritional-value animal proteins, i.e., slow digestive protein (casein) and fast digestive protein (soluble milk proteins).

## 2. Materials and Methods

### 2.1. Raw Material for Pasta Manufacturing

The faba bean (*Vicia faba*), lentil (*Lens culinaris*) and split pea (*Pisum Sativum L*.) flours were supplied by GEMEF Industries (Aix-en-Provence, France), Celnat (Saint-Germain-Laprade, France), and Moulin des Moines (Krautwiller, France), respectively. Panzani provided wheat (*Triticum durum*) semolina (Marseille, France).

### 2.2. Pasta Manufacturing

All pasta were produced at the IATE joint research unit (SupAgro-INRA-Univ Montpellier-CIRAD, Montpellier, France) following the standard pasta-making process steps, i.e., hydration, mixing, and extrusion (40 °C) in a pilot press (Bassano, Lyon, France) and drying at low temperature (55 °C, 15 h) in a pilot drier (AFREM, Lyon, France) [48].

Each pasta was cooked for its optimal cooking time (OCT) + 1 min. to ensure complete starch gelatinization, then dried at low temperature (40 °C, 24 h), and ground down to produce a food material ready for the rats to consume.

Three isoproteic (21% db) legume-enriched pasta were manufactured:

1. A 38% wheat semolina–62% faba bean flour pasta (F-pasta)
2. A 35% wheat semolina–65% lentil flour pasta (L-pasta)
3. A 21% wheat semolina–79% split pea flour pasta (P-pasta)

### 2.3. Diet Manufacturing

Diets were manufactured by the UPAE (Unité de Préparation des Aliments Expérimentaux, INRA, Jouy-en-Josas, France). The diets incorporated the legume-enriched pasta produced by the IATE or casein and soluble milk proteins provided by Lactalis (Torcé, France). Soluble milk proteins, noted as SMP, indicate whey proteins in this present work. Diet compositions (Table 1) were calculated to ensure that both diets that were made with legume-enriched pasta and control diets (casein and SMP) were isoproteic (same protein content) and isocaloric. Dietary amino acid levels (Table 2) were analyzed by the Agrobio laboratory (Rennes, France) according to the method that was published in Commission Regulation (EC) No 152/2009.

### 2.4. Animals and Experimental Protocols

All facilities and procedures were approved by the institution’s animal ethics committee ‘CEMEAA’ (*Comité d’Ethique en Matière d’Expérimentation Animale Auvergne*; approval No. 5535-20160601140512) and they were used in accordance with the European guidelines for the care and use of laboratory animals (2010-63UE). Our research complies also with the commonly-accepted ‘3Rs’ i.e., Replacement, Reduction, and Refinement. All experiments were conducted in such a way as to avoid animal discomfort and minimize animal pain and distress. The study used 43 old (22-month-old) male Wistar rats that were purchased from Janvier (Le Genest-St-Isle, France). The rats were individually housed with free access to water and feed in the research unit’s animal facility (approval No. D6334515), and kept under controlled conditions, i.e., a 12 h light-dark cycle with lights on at 08:00 a.m., and temperature held between 20 °C and 22 °C. 

The rats were randomized into five groups after a week of acclimatization. Two groups were fed a control diet containing casein or SMP (*n* = 9 per group) as sole protein source, and three groups were fed a diet made with F-pasta (*n* = 9), L-pasta (*n* = 8), or P-pasta (*n* = 8) as the sole protein source. All diets were isocaloric and isoproteic (Table 1) and given for six weeks.

Bodyweight and food intake were measured weekly. At the end of the experiment, the rats were anesthetized and sacrificed by exsanguination. The *plantaris*, *soleus*, *tibialis*, *gastrocnemius*, and *quadriceps* muscles were quickly removed, weighed, snap-frozen in liquid nitrogen, and stored at −80 °C. Liver, heart, intestine, and perirenal and subcutaneous adipose tissues were also collected, weighed, snap-frozen in liquid nitrogen, and stored at −80 °C.

### 2.5. Body Composition

The body composition, i.e., fat and lean mass, of non-anesthetized living rats was measured at the beginning, middle (after three weeks) and end (after six weeks) of the study while using an EchoMRI system (Echo Medical Systems, Houston, TX). The rats were fasted for about 12 h before the measurement, but had unlimited access to drinking water. Results are expressed in lean mass gain and fat mass gain. The lean mass gain was calculated as the difference in lean mass between the beginning and the end of the study as a ratio of initial lean mass of the old rats.

### 2.6. Dietary Protein Quality Indexes

For the last four days of the experiment, the rats were placed in metabolic cages with facilities for separating and collecting urine and fecal matter. Urine and feces were collected to quantify excreted nitrogen by the Dumas method [50] at Institut UniLaSalle (Beauvais, France) and to evaluate protein quality parameters. Nitrogen balance, apparent and true protein digestibility, net protein utilization, and biological value were calculated according to the following equations (Equations (1)–(5)); [51]:

(1) NB (g)=NI −(FN + UN)

(2) $$\mathrm{AD}\;(\%)=\frac{\mathrm{NI}\;-\;\mathrm{FN}\;}{\mathrm{NI}}\times 100$$

(3) $$\mathrm{TD}\;(\%)=\frac{\mathrm{NI}\;-\left(\mathrm{FN}\;-\mathrm{EFN}\right)}{\mathrm{NI}}\times 100$$

(4) $$\mathrm{NPU}(\%)\;=\;\frac{\mathrm{NI}\;-\left(\mathrm{FN}\;+\mathrm{UN}\;-\;\mathrm{EFN}\;-\mathrm{EUN}\right)}{\mathrm{NI}}\times 100$$

(5) $$\mathrm{BV}\;(\%)=\frac{\mathrm{NPU}\;}{\mathrm{TD}}\times 100$$

where NB is nitrogen balance, NI is nitrogen intake, FN is fecal nitrogen, UN is urinary nitrogen, AD is apparent digestibility, TD is true digestibility, EFN is endogenous fecal nitrogen, EUN is endogenous urinary nitrogen, NPU is net protein utilization, and BV is biological value.

Fecal and urinary endogenous nitrogen excretions were deduced from a group of old rats that received a nitrogen-free diet during the metabolic cage period.

### 2.7. Biochemical Analyses

Blood glucose level was measured using a Konelab 20 chemical analyzer (Thermo Scientific, MA). Blood insulin level was measured while using an ELISA kit (PromoKine, France) according to the manufacturer’s instructions.

### 2.8. Tissue Protein Extraction and Content

The total proteins were extracted from 50 mg of *plantaris*, *gastrocnemius* and liver in 500 μL of SET extraction buffer (0.25 M Sucrose, 2 mM EDTA, 10 mM Tris, pH 7.4). After homogenization with Mini Beadbeater^®^ (BioSpec Products, Bartlesville, OK, USA), the homogenate was removed, transferred to a hemolysis tube, and then sonicated three times for 10 s at 70% of maximal power (Vibracell 75185, VWR International, Radnor, PA, USA). A colorimetric protein assay was then carried out after protein extraction. The samples and a standard range, made from bovine serum albumin (BSA), were deposited on a 96-well microplate. A bicinchoninic acid reagent from the Micro BCA protein assay kit (Thermo Fischer Scientific, Waltham, MA, USA) was also added. Protein concentration was estimated as BSA equivalent and measured at a 562-nm wavelength while using a microplate reader (Microplate Spectrophotometer Epoch Biotek, Winooski, VT, USA) after incubation for 1 h at 37 °C without agitation. The total protein content in muscle and liver was expressed in mg of proteins. The efficiency of tissue protein anabolism, defined as the ratio of tissue protein content relative to cumulative protein intake over the six weeks diet period, was also calculated as per Mantha et al. [52].

### 2.9. Protein Synthesis Rate in Plantaris Muscle

We measured absolute synthesis rate (ASR) of proteins in *plantaris* muscle using the flooding dose method to evaluate skeletal muscle protein synthesis, as previously described [53]. Briefly, after overnight food deprivation, the rats were infused with 50% excess mol, 300 μM/100 g L(1-13C)valine (Eurisotop Saint-Aubin, France). The tracer uptake time was 50 min. A 50 mg sample of *plantaris* muscle was used for analysis. Proteins were hydrolyzed (6 N HCl, 110 °C, 24 h) then the amino acids were derivatized. L(1-13C)valine enrichment was measured in hydrolyzed proteins while using gas chromatography–combustion–isotope ratio mass spectrometry (Gas System; Fisons Instruments, VG Isotech, Middlewich, UK). L(1-13C)valine enrichment in tissue fluid was evaluated using gas chromatography–mass spectrometry (Hewlett-Packard 5971A; Hewlett-Packard Co., Palo Alto, CA, USA). ASR was calculated according to fractional synthesis rate [53] and total muscle protein content using the following equation (Equation (6)):

ASR (mg/h) = (Ei × 100) × P × M/(Ep × t)(6)

where Ei is enrichment as atom percent excess of L(1-13C)valine derived from valine from proteins at time t (minus basal enrichment), Ep is mean enrichment in the precursor pool (tissue fluid L(1-13C)valine), t is uptake time in hours, P is protein content in muscle in mg per mg of muscle, and M is muscle mass in mg. The data are expressed in mg of total protein synthesis per hour.

### 2.10. Statistical Analysis

Analysis of variance (ANOVA) and Fisher’s protected least significant difference (PLSD) test were used to determine significant differences between the groups. Differences were considered to be significant at *p* < 0.05. Statistical data analysis was performed using StatView^®^ software (SAS, Inc. Intitute, Release 5, 1992-82, Cary, NC, USA).

## 3. Results

### 3.1. Diet Compositions

All of the diets were isocaloric and isoproteic (Table 1). On a whole, the legume-enriched pasta had comparable EAA compositions, except for aromatic amino acid, branched-chain amino acid, and lysine contents that were slightly higher in L-pasta and P-pasta than in F-pasta (Table 2). However, proteins from legume-enriched pasta were characterized by a different amino acid composition as compared to animal-protein diets. Casein and SMP diets had a two-fold higher sulfur amino acid content and 1.2-fold higher branched-chain amino acid content than legume-enriched pasta. Legume-enriched pasta had two-fold higher glycine content and three-fold higher arginine content than casein and SMP diets.

### 3.2. Body Composition

**Figure 1:**
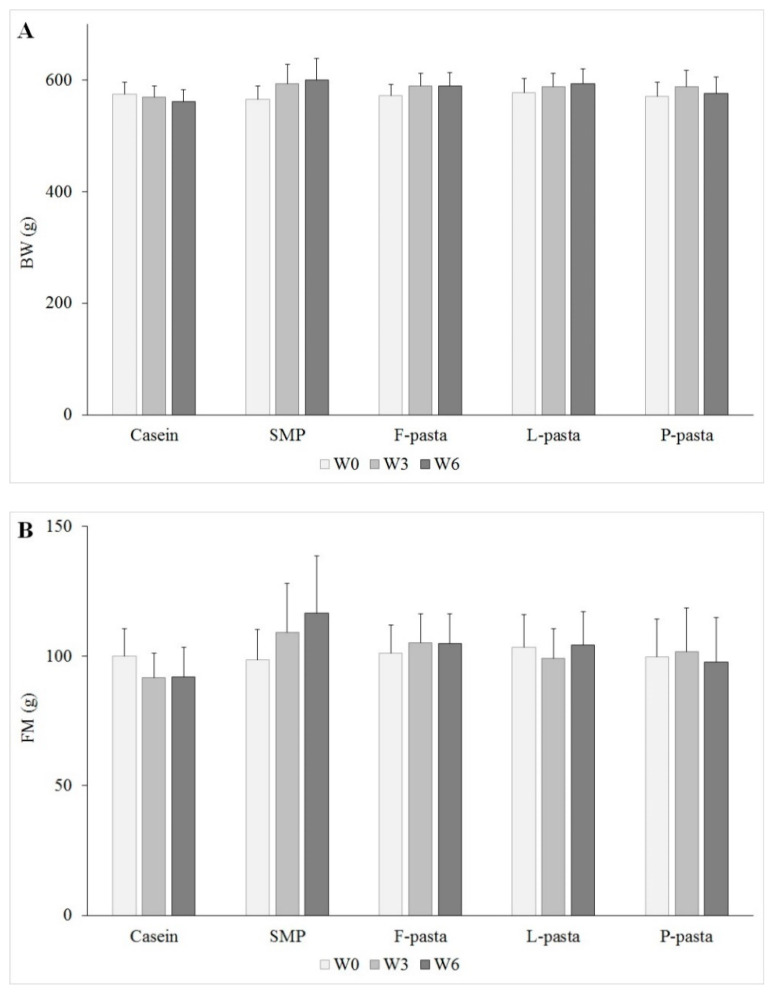

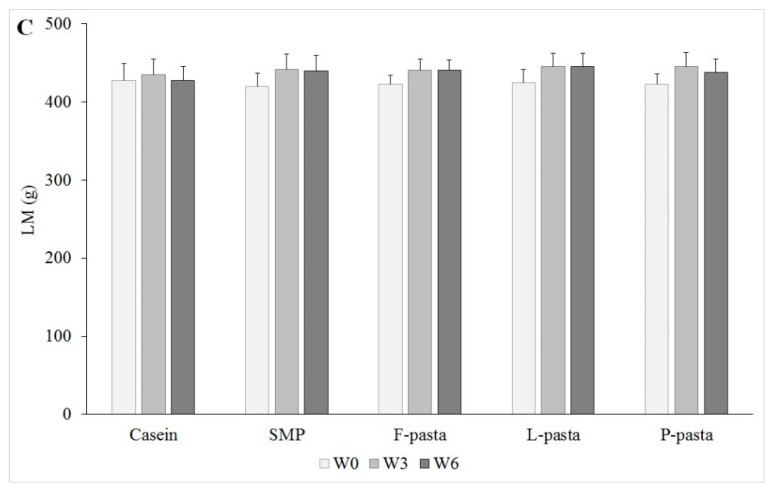
Bodyweight (**A**), fat mass (**B**), and lean mass (**C**) variations during the time. The results are expressed as means ± SEM; Means with different letters are significantly different (*p* < 0.05). W0, W3, and W6 = the beginning, the middle, and the end of the experiment, respectively; BW = bodyweight; FM = fat mass; LM = lean mass; F-pasta = faba bean-enriched pasta; L-pasta = lentil-enriched pasta; P-pasta = split pea-enriched pasta; and, SMP = soluble milk proteins.

Bodyweight, fat mass, and lean mass were not statistically different between diet groups at each point of time i.e., the beginning, the middle, and the end of the experiment. For each diet group, there were no statistical differences with time for bodyweight, fat mass, and lean mass, respectively (Figure 1). Bodyweight, fat mass gain, and lean mass gain were higher in rats that were fed SMP and legume-enriched pasta as compared with rats fed casein (Table 3). In old rats, lean mass was increased under all the diets (by 3.3%–5.1% increase), except for the casein diet (0.3%), but without statistically significant between-group difference (*p* = 0.15). The increase in fat mass tended to be higher in rats fed SMP (14.9 ± 7.8%) than rats fed F-pasta (3.6 ± 3.0%) and L-pasta (1.2 ± 6.4%) diets (*p* = 0.13 and *p* = 0.08, respectively), but was significantly higher than rats fed casein (−8.7 ± 4.3%) and P-pasta (−3.6 ± 3.4%) diets (*p* < 0.05). As a result, after six-week diets, the bodyweight of old rats was increased in legume-enriched pasta and SMP groups, but decreased in the casein group (*p* < 0.05) (Table 3).

### 3.3. Evaluation of Dietary Protein Quality

Table 4 reports protein quality indexes. Food intake calculated during the six-week period was not different between groups (data not shown). Fecal nitrogen content-to-nitrogen intake ratio was 1.4-fold higher in old rats that were fed F-pasta and P-pasta and two-fold higher in old rats fed L-pasta when compared to rats fed casein and SMP diets (*p* < 0.001). The urinary nitrogen content-to-nitrogen intake ratio was similar between rats fed SMP and L-pasta diets, but 29% lower than in rats fed casein and F-pasta diets (*p* < 0.05) and slightly lower than in rats fed P-pasta (−19%, *p* = 0.14).

Nitrogen balance, i.e., the difference between nitrogen intake and nitrogen losses when considering urinary and fecal losses, was higher in rats that were fed the SMP diet as compared to casein (0.79 ± 0.10 g vs. 0.43 ± 0.11 g, respectively; *p* < 0.01). The nitrogen balance was in the same range in rats fed either casein or legume-enriched pasta. Nitrogen balance was not significantly different between rats fed SMP diet when compared to L-pasta (0.68 ± 0.09 g) and P-pasta (0.53 ± 0.09 g; *p* = 0.06).

Apparent digestibility considers all of the digestive processes involving protein digestion, including endogenous nitrogen losses. Apparent digestibility was similar between SMP and casein groups and 5% higher than in the F-pasta and P-pasta groups and 14% higher than in the L-pasta group (*p* < 0.001). True digestibility considers the specific digestion of dietary protein by subtracting endogenous nitrogen losses. Like for apparent digestibility, true digestibility was significantly higher in rats that were fed animal-source proteins, i.e., casein and SMP, than legume-enriched pasta, especially L-pasta (*p* < 0.001).

Net protein utilization (NPU) is the ratio of retained nitrogen to ingested nitrogen. After six weeks of diet, NPU was 39% higher in rats that were fed SMP diet as compared to casein diet (*p* = 0.01). Old rats fed legume-enriched pasta had a similar NPU to the casein group but a lower NPU than the SMP group (*p* < 0.05). Biological value (BV) is the ratio of retained nitrogen to absorbed nitrogen. The BV of SMP (63.9 ± 3.9%) was 37% higher than the BV of casein (46.6 ± 5.7%; *p* = 0.01). Overall, legume-enriched pasta had a similar BV to casein. However, F-pasta had a significantly lower BV than SMP (42.6 ± 5.0%; *p* < 0.01), and P-pasta tended to have a lower BV than SMP (49.7 ± 5.8%; *p* = 0.06), whereas L-pasta had a similar BV to SMP (56.7 ± 4.9%).

### 3.4. Biochemical Analyses

Table 5 provides biochemical analyses. Fasting insulin and glucose levels were not statistically different between groups. Note that, the fasting insulin level tended to be higher in SMP group as compared to legume enriched pasta, i.e., L- and P-pasta groups, and casein group (*p* = 0.07).

### 3.5. Tissue Weight, Protein Content, and Protein Anabolism Efficiency

Table 6 reports tissue weights. As expected, total skeletal muscle mass (the sum of *plantaris*, *soleus*, *tibialis*, *gastrocnemius,* and *quadriceps* weights) tended to be higher in old rats fed SMP than those fed casein (*p* = 0.07). Rats that were fed legume-enriched pasta had similar skeletal muscle mass to rats fed the casein diet and rats fed L-pasta and P-pasta even had similar skeletal muscle mass to rats fed the SMP diet. For example, the *plantaris* muscle mass was higher in the SMP group (428 ± 23 mg) when compared to the casein group (341 ± 28 mg) and F-pasta group (329 ± 35 mg), but was similar in the SMP group as compared to the P-pasta (366 ± 19 mg) and L-pasta (390 ± 17 mg) groups (*p* = 0.07). There were no significant between-group differences in liver weight (*p* = 0.57), adipose tissue weight (the sum of subcutaneous and perirenal adipose tissues) (*p* = 0.80), and intestine weight (*p* = 0.98).

Tissue protein contents and protein anabolism efficiency were determined for *plantaris* and *gastrocnemius* muscles and for liver (Table 7). The protein content in *plantaris* muscle was higher in the SMP group (50.33 ± 4.66 mg proteins) as compared to rats fed casein (32.43 ± 4.24 mg proteins) and F-pasta group (35.97 ± 4.58 mg proteins) and similar between the P-pasta group (38.57 ± 2.99 mg proteins) and the L-pasta group (46.03 ± 6.57 mg proteins). Protein anabolism efficiency in *plantaris* and *gastrocnemius* muscles was similar between rats that were fed legume-enriched pasta and fed casein or SMP (*p* > 0.05).

The liver protein content was higher in the P-pasta group (3.12 ± 0.12 g) than in the casein and SMP groups (2.45 ± 0.11 and 2.58 ± 0.18 g). Consequently, liver protein anabolism efficiency was higher in the P-pasta group (2.4 ± 0.1%) than the casein and SMP groups (2.0 ± 0.2 and 1.8 ± 0.1%, respectively).

### 3.6. Absolute Protein Synthesis Rate in Plantaris Muscle

**Figure 2:**
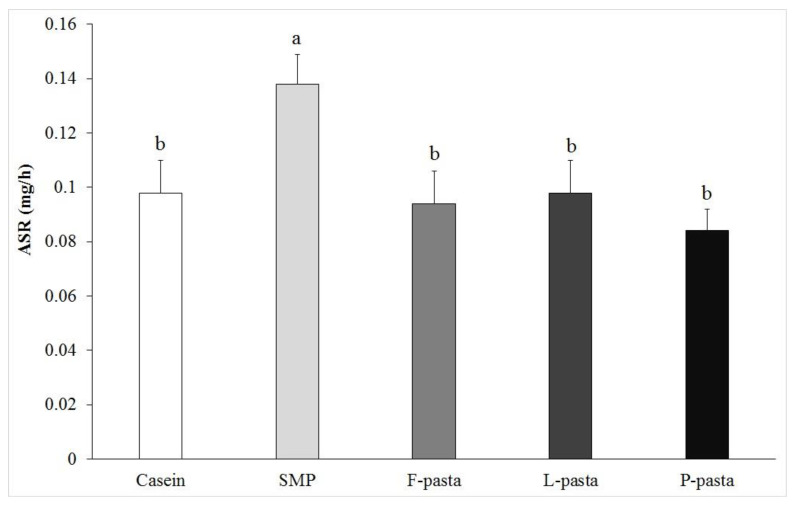
Protein synthesis rate in *plantaris* muscle in old rats after six weeks of diet; results are expressed as means ± SEM; means with the same letters are not significantly different (*p* > 0.05). ASR = absolute synthesis rate; F-pasta = faba bean-enriched pasta; L-pasta = lentil-enriched pasta; P-pasta = split pea-enriched pasta; SMP = soluble milk proteins.

ASR was measured in *plantaris* muscle (Figure 2). As expected, ASR was significantly higher (+41%) in the SMP group when compared to the casein group (0.138 ± 0.011 mg/h vs. 0.098 ± 0.012 mg/h, respectively; *p* < 0.05). Moreover, ASR in all legume-enriched pasta groups, i.e., F-pasta (0.094 ± 0.012 mg/h), L-pasta (0.098 ± 0.012 mg/h) and P-pasta (0.084 ± 0.008 mg/h), was similar to the casein group, but significantly lower, by 29%–39%, than for SMP (*p* < 0.05).

## 4. Discussion

The aim of this study was to evaluate the protein quality of wheat pasta enriched with faba bean or lentil or split pea flour and the impact of their intake on protein retention and metabolism in old rats as compared to dietary animal proteins, i.e., casein and SMP. The nutritional value of proteins is dependent on their amino acid composition and how readily they can be digested, absorbed, and incorporated into body proteins [54]. As expected, casein and SMP had higher apparent and true protein digestibility than legume-enriched pasta, especially the L-pasta. Sarwar et al. [55] noted that the presence of residual anti-nutritional factors, i.e., trypsin inhibitors, lectins, amylase inhibitors, in cooked pulses could interfere with protein digestion and increase endogenous protein excretion, particularly proteases, when compared to animal proteins [56]. In old rats, this could result in increased fecal nitrogen excretion and lower protein digestibility for legume-enriched pasta as compared to animal protein-based diets. Here, we found that protein digestibility differed between source-pulses. Specifically, the protein digestibility of F- and P-pasta was higher than that of L-pasta. Accordingly, the residual activity of trypsin inhibitors was 5% and 21% higher in L-pasta than in F- and P-pasta, respectively [57]. Protein digestibility might also be affected by protein aggregation through steric hindrance or even by protein covalent crosslinking, which limits proteolytic enzyme access and action with and towards peptide bonds [58]. L-pasta contained 11% and 14% higher proportions of covalently-linked proteins, i.e., disulfide-bonded proteins rather than weakly-linked proteins, than F- and P-pasta, respectively [57].

Higher protein digestibility promotes a higher amount of amino acid available for absorption and, thus, greater nutritional value [59]. NPU was equivalent between old rats fed legume-enriched pasta or casein despite a lower digestibility of legume-enriched pasta proteins. Urinary nitrogen excretion in old rats was lower after L-pasta intake and, to a lower extent, after P- and F-pasta intake than casein, leading to an equivalent nitrogen retention in the body. This contrasts with Löhrke et al. [60], who reported a higher urinary nitrogen excretion and increased plasma level of urea in pigs in response to dietary soybean protein isolate when compared to casein. Laleg et al. [46] demonstrated that protein utilization increased by 75% in young rats fed wheat pasta enriched with 35% faba bean flour as compared to an isoproteic wheat pasta enriched with gluten, but remained 9% lower than in rats fed casein. Mixed wheat–legume pasta has a more balanced amino acid composition when compared to wheat protein alone, i.e., gluten-enriched pasta, which could explain the higher protein utilization [46]. Furthermore, contrary to what was shown with the 35% faba bean pasta [46], here we observed equivalent protein utilization between legume-enriched pasta and casein diets when the legume enrichment level in the pasta increased up to 62–79%. The essential amino acid composition of the legume-enriched pasta used here was closer to casein and to rat needs according to National Research Council [61] than for the 35% faba bean pasta [46]. However, here, the measured net protein utilization was lower in old rats fed casein or legume-enriched pasta than SMP. Note that this difference could be explained not only by the essential amino acid composition, in particular a higher leucine content (+51% as compared to legume-enriched pasta), but also to the rapid assimilation of SMP [62].

Fat mass gain and lean mass gain, in particular muscle mass change, were equivalent in these old rats that were fed the different legume-enriched pasta after six weeks of diets, regardless of the legume flour used. Therefore, there was no effect of legume type and, more precisely, of legume/wheat protein ratio on body composition in old rats. In addition, old rats fed legume-enriched pasta had comparable lean mass gain to rats fed casein or SMP, regardless of pulse source. Furthermore, the weight and protein content of *plantaris* and *gastrocnemius* muscles were grossly similar between the rat groups fed legume-enriched pasta and the casein and SMP groups. Several studies have evaluated the effect of dietary protein sources on protein metabolism, but were generally done in young rats. Wróblewska et al. [35] evaluated the body composition of young rats that were fed a commercial soy protein preparation and demonstrated that they had a lower bodyweight, fat and lean mass gain than rats fed a whey protein preparation. Martínez and Larralde [18,19] demonstrated that feeding young rats with diets containing raw legume, i.e., *Vicia faba,* induced a significant decrease in muscle mass compared to casein. However, the authors did not find any difference in myofibrillar protein breakdown between rats receiving raw legume and casein. They suggested that the decrease in muscle mass could be explained by a decrease in muscle protein synthesis in rats that were fed raw legume due to the presence of antinutritional compounds, like trypsin inhibitors in raw *Vicia faba* that reduce protein digestibility. Here, the manufacturing process used, including cooking, significantly decreased the activity of trypsin inhibitors as compared to raw flours and resulted in a drastic reduction of trypsin inhibitors in the legume-enriched pasta, i.e., a residual activity of 3%–5% in comparison to the raw legume flour [57]. Alonso et al. [25] found a lower muscle mass and muscle protein content in young rats fed seed peas deficient in certain essential amino acids, even if extruded and cooked to reduce antinutritional factor content, than after casein intake. Taken together, the data that are presented here showed that mixed wheat–legume pasta intake promoted a similar muscle gain to casein intake and even SMP intake (for L- and P-pasta) in old rats. Mixing wheat and legume flour in pasta also resulted in more efficient muscle mass maintenance than when using legumes [18,19,25]. Combining wheat and legume proteins in pasta allowed for a needs-balanced amino acid intake, which is vital for maintaining or even increasing muscle mass, in particular in old rats. The capacity of legume-enriched pasta to increase lean mass, in particular muscle mass, could be beneficial for older people that are exposed to sarcopenia.

The changes that were observed for protein synthesis rates in *plantaris* muscle in old rats were relatively in line with changes in muscle mass and muscle protein content. Muscle mass and protein content tended to be higher in the SMP group as compared to the casein and F-pasta groups, although not compared to L- and P-pasta groups. We suggest that L- and P-pasta intake could enhance postprandial muscle protein anabolism in old rats, which would translate into muscle protein accretion and increased muscle mass. Several studies have compared the influence of plant-based proteins and animal-based proteins on muscle protein synthesis. In young rats, the protein synthesis rate was lower in *gastrocnemius* muscle [21] after raw faba bean intake than after milk protein intake. In addition, young rats had a lower muscle protein synthesis rate when fed cooked beans and lentils when compared to casein [24], although the protein synthesis rate was higher in the large and small intestine [24,26]. In older humans, Yang et al. [37] showed that the muscle protein synthesis rate was lower in people fed soy protein isolate than those fed whey protein isolate in both rested and post-exercise conditions. A more recent study demonstrated that the muscle protein synthesis rate in older people was lower after consumption of 35 g of wheat protein hydrolysate as compared to an equal amount of casein [38].

Taken together, these studies showed that, in young as well as in old individuals, the muscle protein synthesis rate was higher after milk protein intake when compared to legume or wheat protein consumed alone. Interestingly, we clearly demonstrated here that the muscle protein synthesis rate was not different in old rats fed casein when compared to counterparts fed a mixed wheat–legume pasta, although it remained lower than in animals that were fed SMP. The high anabolic effect of SMP (rapidly-digested and leucine-rich protein; [63,64,65]) could explain the ability of this protein to enhance body protein retention and muscle protein synthesis in old rats. Note that this high anabolic effect of SMP resulted in a slight increase in muscle mass together with an increase in muscle protein content as compared to casein and faba bean-enriched pasta. Combe et al. [66] suggested that lysine and arginine from faba bean seeds would be only partially available for protein synthesis in peripheral tissues, e.g., skeletal muscles, as they demonstrated that lysine and arginine contents in muscle were higher in rats fed lentil (*Lens escilenta*) or chickpea (*Cicer arietinum*) than those fed faba bean (*Vicia faba*).

In addition to the balanced essential amino acid composition, the arginine and glycine contents were higher in legume-enriched pasta than casein and SMP diets. These amino acids are known to influence protein synthesis [67,68,69,70]. Several studies have demonstrated that dietary L-arginine supplementation enhances skeletal muscle mass and muscle protein synthesis in rats or pigs [67,68]. This particular effect could be dependent on an increased phosphorylation rate of the Akt/mTOR signaling pathway, which is a key driver of protein synthesis [67,68]. Moreover, the abundance of glycine in legume-enriched pasta is also one of the potential mechanisms leading to enhanced skeletal muscle protein anabolism in old rats. Hence, glycine could increase protein synthesis by Akt/mTOR activation and prevent protein degradation by inhibiting proteolytic gene expression [69].

Like amino acids, insulin plays a major role in promoting postprandial protein anabolism, mainly by inhibiting protein degradation and activating postprandial protein synthesis [71,72]. In post-absorptive conditions, the fasting insulin and glucose levels were not statistically different between groups. Nevertheless, the postprandial insulin level could be different in old rats, according to the diet composition. We suggest that legume-enriched pasta containing gelatinized starch could stimulate insulin secretion [73] when compared to rats fed casein and SMP containing native starch. A cumulative effect of increased insulin secretion, arginine, and glycine contents with better balanced amino acid composition could contribute to the equivalent muscle protein accretion in rats fed legume-enriched pasta as compared to rats fed casein and SMP, in particular for L- and P-pasta. This could partly explain the positive effect of legume-enriched pasta, despite their lower digestibility and lower leucine content, when compared to dietary animal proteins. Legume-enriched pasta could help to overcome this process by providing a balanced amino acid composition and stimulating insulin secretion, which are the two main factors implicated in enhancing meal-related muscle protein anabolism, as aging is associated with impaired muscle anabolic response to food intake [71].

This study assessed protein synthesis at muscle level, but it could also be informative for further research to assess protein distribution in other tissues and evaluate hepatic and intestinal protein synthesis rates. We measured liver protein content and liver anabolism efficiency in old rats. Interestingly, these parameters were higher in the P-pasta group and they tended to be higher in the F-pasta group when compared to groups that were fed animal proteins. Note that, Márquez-Mota et al. [42] demonstrated in young rats that a combination of legume and cereal proteins, i.e., soy and corn protein isolates, significantly enhanced the hepatic Akt/mTOR-signaling pathway when compared to soy protein or corn protein alone and to casein, possibly through stimulation of protein synthesis.

It would have been interesting to add another control group fed a pasta containing only wheat proteins to our research. This experimental design would have evaluated the benefit of mixing legume protein sources with wheat protein sources to compensate for the low anabolic effect of each plant protein alone. Nevertheless, the outcome of this group was expected. Impaired food efficiency, muscle mass, and nitrogen retention were observed in young rats that were fed wheat pasta enriched with gluten as compared to legume-enriched pasta group, according to previous data, as discussed above [46]. Feeding rats with diets deficient in lysine alter the nitrogen balance of animals [43,44,45,46]. Therefore, rat group fed only wheat proteins was not added in our research in order to avoid observing the consequences of lysine deficiency of cereal proteins. The comparison of our study results with those reported in the literature [43,44,45,46] led us to conclude that the enrichment of wheat pasta with legume protein sources could compensate the lower anabolic effect of wheat proteins and improve their nutritional quality, especially in old rats.

## 5. Conclusions

We demonstrated that net protein utilization in old rats fed legume-enriched pasta diets was similar to those fed a casein diet and lower than with an SMP diet. At the tissue level, legume-enriched pasta intake led to significant protein accretion in the liver. In addition, legume-enriched pasta intake induced a similar muscle protein synthesis rate to a casein diet, but a lower rate than a SMP diet. Moreover, legume enriched pasta intake led to an equivalent effect on muscle weight and muscle protein accretion in old rats when compared to casein and SMP. However, the muscle protein synthesis rate was higher in rats fed the legume-enriched pasta than in animals fed each legume source alone (based on data from previous studies). Blending wheat and legume in a staple food, like pasta, led to improve its essential amino acid profile that is more adequate for muscle synthesis rate and muscle protein accretion especially for older individuals. These new food matrices combining wheat and legume flours have an equivalent nutritional quality to that of casein regardless of the legume type used, but still lower than that of soluble milk proteins “whey proteins”.

## Figures and Tables

**Table 1:** Chemical composition of legume-enriched pasta and experimental diets.

Pasta Composition			F-Pasta	L-Pasta	P-Pasta
**Protein (%, db)**			21.2	21.2	21.5
**Total starch (%, db)**			64.4	59.1	58.5
**Total fiber (%, db)**			7.4	11.7	10.4
**Lipids (%, db)**			1.4	1.6	2.6
**Diet ingredients (g)**	**Casein**	**SMP**	**F-Pasta**	**L-Pasta**	**P-Pasta**
**Casein**	18.2				
**SMP**		18.2			
**F-pasta**			90		
**L-pasta**				90	
**P-pasta**					90
**Native starch (corn)**	64.7	64.7	1.2	1.2	1.2
**Cellulose**	6.6	6.6			
**Lipids (soy)**	6	6	4.3	4.3	4.3
**Minerals**	3.5	3.5	3.5	3.5	3.5
**Vitamins**	1	1	1	1	1
**Total diet ingredients**	100	100	100	100	100
**Diet composition (wt%)**	**Casein**	**SMP**	**F-Pasta**	**L-Pasta**	**P-Pasta**
Protein (%) *	16.4	16.4	16.7	16.8	17.0
Carbohydrate (%) **	61.6	61.6	57.9	57.1	55.5
Lipid (%) ***	6	6	5.4	5.6	6.4
**Energy (Kcal/100 g)**	366	366	347	346	348

F-pasta = faba bean-enriched pasta; L-pasta = lentil-enriched pasta; P-pasta = split pea-enriched pasta; SMP = soluble milk proteins; wt% = percentage by weight (%, wt/wt); db = dry basis; * Protein content in the diet was calculated from 90 g of legume-enriched pasta and determined using the Kjeldahl method (NF V 03–050, 1970). ** Carbohydrate contents of legume-enriched pasta diets are calculated from the carbohydrate added to the diet, i.e., native corn starch, and carbohydrate provided from legume-enriched pasta, i.e., total starch, determined while using an enzymatic assay kit (Megazyme, Co. Wicklow, Ireland; AACC method 76–13.01) and total fiber determined according to AOAC 991-42 (for soluble fiber) and AOAC 993-19 (for insoluble fiber) methods. *** Lipid contents of legume-enriched pasta diets were calculated from the lipid added to the diet, i.e., soy oil, and the lipids provided by legume-enriched pasta determined according to French government paper 08-09-1977.

**Table 2:** Amino acid composition of the diets.

	Amino Acid Content (mg/g Protein) ^1^				
	Animal Proteins		Legume-Enriched Pasta		
	Casein	SMP ^2^	F-Pasta	L-Pasta	P-Pasta
Aspartic acid	66.9	113.7	97.3	102.8	109.1
Threonine	39.5	50.5	37.0	37.2	38.3
Serine	51.2	45.1	53.1	54.9	52.4
Glutamic acid	213.8	171.4	223.5	215.8	202.4
Proline	102.5	46.0	63.9	61.0	54.2
Glycine	17.7	19.8	42.5	41.0	43.3
Alanine	28.9	47.8	42.0	42.0	44.3
Valine	65.9	51.4	48.2	49.7	49.5
Sulfur AA	45.7	51.4	26.3	26.1	26.0
Aromatic AA	88.2	72.2	74.1	81.4	83.4
Isoleucine	48.5	50.5	43.1	45.2	45.5
Leucine	87.7	120.3	79.1	80.3	78.9
Lysine	74.9	96.5	54.0	57.2	66.9
Histidine	26.1	20.0	26.9	25.3	25.1
Arginine	31.9	22.6	80.0	72.8	73.9
Tryptophan	10.6	20.7	9.0	7.5	7.1
**BCAA**	202.1	222.2	170.4	175.2	173.9
**EAA**	487.1	533.5	397.7	409.9	420.7

^1^ Amino acid composition of casein and legume-enriched pasta diets was determined by Agrobio (Rennes, France) according to the method published in Commission Regulation (EC) No 152/2009. ^2^ Amino acid composition of SMP was calculated from [49]; F-pasta = faba bean-enriched pasta; L-pasta = lentil-enriched pasta; P-pasta = split pea-enriched pasta; AA = amino acid; Sulfur AA = methionine + cysteine; Aromatic AA = tyrosine + phenylalanine; BCAA = branched-chain amino acid; EAA = essential amino acid; SMP = soluble milk proteins.

**Table 3:** Body composition of old rats after six weeks of diet.

Diets	Casein	SMP	F-Pasta	L-Pasta	P-Pasta
∆BW (%)	−2.3 ± 1.8 ^b^	5.6 ± 2.6 ^a^	2.9 ± 1.4 ^a^	2.7 ± 1.5 ^a^	0.9 ± 0.8 ^ab^
∆FM (%)	−8.7 ± 4.3 ^b^	14.9 ± 7.8 ^a^	3.6 ± 3.0 ^ab^	1.2 ± 6.4 ^ab^	−3.6 ± 3.4 ^b^
∆LM (%)	0.3 ± 1.7	4.5 ± 1.3	4.4 ± 1.6	5.1 ± 1.0	3.3 ± 1.3

Results are expressed as means ± SEM. Means sharing the same letters i.e., ^a^ or ^b^, within a line are not significantly different (*p* > 0.05). F-pasta = faba bean-enriched pasta; L-pasta = lentil-enriched pasta; P-pasta = split pea-enriched pasta; SMP = soluble milk proteins; ∆BW = bodyweight gain; ∆FM = fat mass gain; ∆LM = lean mass gain.

**Table 4:** Dietary protein quality evaluation using the nitrogen balance method in old rats after 6 weeks of diet.

Diets	Casein	SMP	F-Pasta	L-Pasta	P-Pasta
FN/NI	0.11 ± 0.01 ^c^	0.09 ± 0.01 ^c^	0.14 ± 0.01 ^b^	0.21 ± 0.02 ^a^	0.14 ± 0.01 ^b^
UN/NI	0.67 ± 0.06 ^a^	0.47 ± 0.04 ^b^	0.65 ± 0.05 ^a^	0.47 ± 0.04 ^b^	0.58 ± 0.05 ^ab^
NB (g)	0.43 ± 0.11 ^b^	0.79 ± 0.10 ^a^	0.42 ± 0.08 ^b^	0.68 ± 0.09 ^ab^	0.53 ± 0.09 ^ab^
AD (%)	88.83 ± 0.59 ^a^	91.26 ± 0.70 ^a^	86.15 ± 0.97 ^b^	79.19 ± 1.68 ^c^	86.13 ± 0.67 ^b^
TD (%)	97.61 ± 0.75 ^a^	98.67 ± 0.74 ^a^	92.85 ± 0.97 ^b^	85.44 ± 1.90 ^c^	93.02 ± 0.84 ^b^
NPU (%)	45.44 ± 5.62 ^b^	63.21 ± 4.15 ^a^	39.23 ± 4.24 ^b^	48.67 ± 4.72 ^b^	46.16 ± 5.27 ^b^
BV (%)	46.60 ± 5.69 ^b^	63.92 ± 3.90 ^a^	42.56 ± 4.99 ^b^	56.69 ± 4.94 ^ab^	49.72 ± 5.81 ^ab^

Results are given as means ± SEM. Means sharing the same letters i.e., ^a, b^ or ^c^, within a line are not significantly different (*p* > 0.05). F-pasta = faba bean-enriched pasta; L-pasta = lentil-enriched pasta; P-pasta = split pea-enriched pasta; SMP = soluble milk proteins; NI = nitrogen intake; FN = fecal nitrogen; UN = urinary nitrogen; NB = nitrogen balance; AD = apparent digestibility; TD = true digestibility; NPU = net protein utilization; BV = biological value.

**Table 5:** Fasting insulin and glucose levels in old rats after 6 weeks of diet.

Diets	Casein	SMP	F-Pasta	L-Pasta	P-Pasta
Insulin (ng/mL)	0.55 ± 0.06	0.85 ± 0.19	0.74 ± 0.12	0.50 ± 0.04	0.51 ± 0.06
Glucose (g/L)	1.29 ± 0.06	1.37 ± 0.05	1.24 ± 0.06	1.26 ± 0.08	1.26 ± 0.06

Results are given as means ± SEM. Means with different letters within a line are significantly different (*p* < 0.05). F-pasta = faba bean-enriched pasta; L-pasta = lentil-enriched pasta; P-pasta = split pea-enriched pasta; SMP = soluble milk proteins.

**Table 6:** Tissue weights in old rats after six weeks of diet.

Diets Tissues	Casein	SMP	F-Pasta	L-Pasta	P-Pasta
**Initial BW**	576 ± 21	566 ± 24	573 ± 20	578 ± 25	571 ± 26
**Final BW**	562 ± 21	600 ± 39	590 ± 24	594 ± 26	577 ± 29
*Plantaris* (mg)	341 ± 28 ^b^	428 ± 23 ^a^	329 ± 35 ^b^	390 ± 17 ^ab^	366 ± 19 ^ab^
*Soleus* (mg)	207 ± 9	249 ± 10	218 ± 19	233 ± 14	220 ± 11
*Tibialis* (mg)	595 ± 51 ^c^	773 ± 35 ^a^	601 ± 67 ^bc^	745 ± 49 ^ab^	703 ± 48 ^abc^
*Gastrocnemius* (g)	1.6 ± 0.1 ^b^	2.0 ± 0.1 ^a^	1.6 ± 0.2 ^b^	1.9 ± 0.1 ^ab^	1.8 ± 0.1 ^ab^
*Quadriceps* (g)	2.9 ± 0.2 ^c^	3.7 ± 0.2 ^a^	3.1 ± 0.3 ^bc^	3.6 ± 0.1 ^ab^	3.2 ± 0.2 ^abc^
**TMM (g)**	11.4 ± 0.6 ^b^	14.3 ± 0.7 ^a^	11.7 ± 1.2 ^b^	13.5 ± 0.5 ^ab^	12.7 ± 0.8 ^ab^
**Heart (g)**	1.60 ± 0.05	1.78 ± 0.04	1.67 ± 0.05	1.75 ± 0.07	1.66 ± 0.05
**Liver (g)**	11.9 ± 0.5	12.7 ± 0.7	13.1 ± 0.7	12.4 ± 0.8	13.2 ± 0.4
**AT (g)**	17.2 ± 2.2	21.9 ± 4.4	20.5 ± 2.7	18.9 ± 2.4	18.2 ± 2.4
**Intestine (g)**	5.5 ± 0.4	5.6 ± 0.4	5.3 ± 0.3	5.4 ± 0.3	5.5 ± 0.4

Results are given as means ± SEM. Means sharing the same letters i.e., ^a, b^ or ^c^, within a line are not significantly different (*p* > 0.05). BW = bodyweight; F-pasta = faba bean-enriched pasta; L-pasta = lentil-enriched pasta; P-pasta = split pea-enriched pasta; SMP = soluble milk proteins; TMM = total skeletal muscle mass, AT = adipose tissue (the sum of perirenal and subcutaneous adipose tissues).

**Table 7:** Tissue protein content and protein anabolism efficiency in old rats after six weeks of diet.

	Casein	SMP	F-Pasta	L-Pasta	P-Pasta
***Gastrocnemius***					
**Total protein content, mg**	230.7 ± 19.4	310.2 ± 33.8	252.2 ± 55.8	273.3 ± 18.7	272.8 ± 17.9
**Protein anabolism efficiency (%)**	0.183 ± 0.014	0.223 ± 0.025	0.196 ± 0.027	0.205 ± 0.016	0.205 ± 0.017
***Plantaris***					
**Total protein content, mg**	32.43 ± 4.24 ^b^	50.33 ± 4.66 ^a^	35.97 ± 4.58 ^b^	46.03 ± 6.57 ^ab^	38.57 ± 2.99 ^ab^
**Protein anabolism efficiency (%)**	0.026 ± 0.003	0.036 ± 0.003	0.029 ± 0.004	0.033 ± 0.005	0.029 ± 0.003
**Liver**					
**Total protein content, mg**	2452 ± 105 ^b^	2578 ± 182 ^b^	2900 ± 142 ^ab^	2717 ± 287 ^ab^	3117 ± 117 ^a^
**Protein anabolism efficiency (%)**	2.00 ± 0.16 ^b^	1.84 ± 0.11 ^b^	2.18 ± 0.11 ^ab^	1.99 ± 0.17 ^b^	2.40 ± 0.09 ^a^

Results are given as means ± SEM. Means sharing the same letters i.e., ^a^ or ^b^, within a line are not significantly different (*p* > 0.05). F-pasta = faba bean-enriched pasta; L-pasta = lentil-enriched pasta; P-pasta = split pea-enriched pasta; SMP = soluble milk proteins.

## Abbreviations

AA	amino acid
AD	apparent digestibility
ASR	absolute synthesis rate
AT	adipose tissue
BCAA	branched-chain amino acid
BV	biological value
BW	bodyweight
EAA	essential amino acid
EFN	endogenous fecal nitrogen
EUN	endogenous urinary nitrogen
F-pasta	faba bean-enriched pasta
FM	fat mass
FN	fecal nitrogen
L-pasta	lentil-enriched pasta
LM	lean mass
NB	nitrogen balance
NI	nitrogen intake
NPU	net protein utilization
P-pasta	split pea-enriched pasta
SMP	soluble milk proteins
TD	true digestibility
TIA	trypsin inhibitor activity
TMM	total skeletal muscle mass
UN	urinary nitrogen
